# Arsenic in Water Resources of the Southern Pampa Plains, Argentina

**DOI:** 10.1155/2009/216470

**Published:** 2009-08-25

**Authors:** Juan D. Paoloni, Mario E. Sequeira, Martín E. Espósito, Carmen E. Fiorentino, María del C. Blanco

**Affiliations:** ^1^Consejo Nacional de Investigaciones Científicas y Técnicas (CONICET), San Andrés 850, 8000 Bahía Blanca, Argentina; ^2^Departamento de Ingeniería, Universidad Nacional del Sur. Consejo Nacional de Investigaciones Científicas y Técnicas (CONICET-CERZOS), Avenida Alem 1253, 8000 Bahía Blanca, Argentina; ^3^Departamento de Agronomía, Universidad Nacional del Sur, San Andrés 850, 8000 Bahía Blanca, Argentina

## Abstract

Confronted with the need for accessible sources of good quality water and in view of the fact that the threat to public health posed by arsenic occurs mainly through the ingestion of contaminated drinking water, the presence and distribution of arsenic was evaluated in the southern Pampa Plains of Bahía Blanca district in Argentina. The findings show variable concentrations of arsenic in a complex distribution pattern. Complementary information is provided on the behavior of the groundwater resource and its salinity in terms of dissolved ions. Groundwater is the most severely affected, 97% of the samples exceeding the guideline value for arsenic in drinking water as recommended by the WHO (Guidelines for Drinking Water Quality, 2004). and showing maximum concentrations of up to 0.30 mg/L. Informing those responsible for preventive medicine and alerting the community at large will facilitate measures to mitigate exposure and ensure the safety of drinking water.

## 1. Introduction

The need to provide safe and accessible supplies of water to cover at least the basic requirements of the world's growing population is increasingly becoming a concern not only for international organizations dealing with this subject but also for policy-makers responsible for meeting demand in those regions most at risk from chronic water shortage [1]. 

Considerable literature has been published in recent years reporting the detection of arsenic in groundwater used for human consumption in new areas around the globe [2–6] and particularly in Argentina [7–9], where research on this etiologic agent of hydroarsenicism dates back to the early 20th century [10]. 

The consumption of contaminated water over long periods of time is the primary route of human exposure to arsenic [11]. However, medical toxicologists lack basic information on the presence and distribution of this ion in drinking water, thus hindering their ability to take preventive action and restricting them to the treatment of those already affected. Lesions to the skin, skin cancer, various other types of cancer (lung, kidney, liver, bladder), peripheral neuropathology's, and vascular pathologies (such as blackfoot disease) are all health problems commonly associated with long-term drinking of arsenic-contaminated water. 

In view of its toxicity and the large number of people exposed to its effects worldwide, arsenic is an environmental contaminant that imposes a high risk of morbidity and mortality [12]. It is highly likely that in many as yet untested areas of Argentina, the population is drinking water with excessive concentrations of arsenic. The more studies undertaken on a country's water resources, the more evidence there is of the presence of this contaminant. According to Hopenhayn [12] limited data are available regarding the extent of arsenic contamination high-risk areas of Argentina. Thus, because of the delayed onset of illness, forty years or more may pass from the time of exposure until hydroarcenicism is diagnosed.

The present study was motivated by the high concentrations of arsenic found in groundwater of the southwest of the province of Buenos Aires, Argentina [13–15], and aims at evaluating arsenic concentrations, determining the spatial distribution of this contaminant in the southern Pampa Plains of Bahía Blanca district, and ultimately gauging the incidence risk in the environment of the local community, which has the region's highest population density.

## 2. Materials and Methods

**Figure 1:**
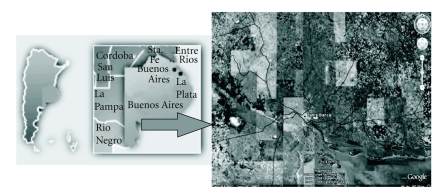
Southern Pampa Plains and Bahía Blanca, Argentina—Geographical location.

The study was carried out over an area of 2 300 km^2^ in the southern Pampa Plains of Argentina within the Bahía Blanca district, which has a population of approximately 320 000 and borders on the Atlantic coast. The coordinates of the city of Bahía Blanca are Latitude 38°44′ S and Longitude 62°16′ W of Greenwich (Figure 1).

Undulating plains and a moderate climate characterize the area, the main economic activities being in the agriculture and livestock sectors, with a considerable and stable resident rural population. Farming and ranching activities have impinged heavily on water and soil resources over the last century. These activities have gradually degraded resources in the absence of sustainable management techniques—despite some limited attempts at conservationism—and in the face of intensified anthropic use. The economy of the region depends in great measure on the quantity and quality of accessible water. 

**Figure 2:**
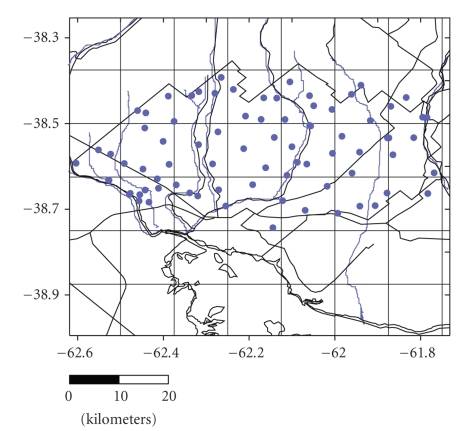
Map of surface and groundwater sampling sites.

The cartographical information used for the study is based on maps of the Instituto Geográfico Militar (Military Geographical Institute), scales 1 : 50,000 and 1 : 100,000, and Landsat rural maps and images. A total of 81 water samples and three replicates were gathered through selective sampling in wells and perforations in use and in streams and minor water courses (Figure 2). Groundwater was extracted by means of windmills and centrifuge pumps, or similar. The water samples were collected in sterile 500 mL polyethylene containers and after filtration were subjected to the following analyses: pH (potentiometer), electrical conductivity (conductimeter), hardness, dissolved solids (evaporation), calcium+magnesium (versenate method), calcium, sodium and potassium (photometry), magnesium (EDTA complexometry), sulfates (turbidimetry), carbonates and bicarbonates (volumetry with sulfuric acid), nitrates (specific electrodes), and phosphates. However, according to our objective, here in solely electrical conductivity, water table depth and arsenic concentrations in phreatic and surface waters are reported (Tables 1a, 1b, and 1c). The depth and temperature of the water were recorded for all samples.

The concentration of arsenic in water was determined by Hydride Generation and Inductively Coupled Plasma Emission and Inductively Coupled Plasma Atomic Emission Spectroscopy (ICP-AES; detection limit 0.33 ppb) based on the method of [16], involving the continuous generation of arsenic (AsH_3_) using three of the four channels of a peristaltic pump (Cole Palmer Instruments Co, Masterflex). The sample solution and a solution of sodium tetrahydroborate plus potassium iodate were transported to a modified liquid-gas separator. The arsine and the hydrogen were captured from the separator to the plasma by a continuous flow of argon. Arsenic was determined according to the length of its main wave at 193.69 nm. Boron, fluoride, chrome, vanadium, and other toxic elements were analyzed by Inductively Coupled Plasma (ICP). The chemical analyses were subjected to quality control through standard solutions for ICP; an Aldrich solution for arsenic was used as a reagent blank to obtain calibration curves for quality assurance being the analytical better than 5%. 

The hydrodynamics of the phreatic levels are presented in an isohypsic chart and the geographical distribution of total soluble salt concentrations and of arsenic are given in isoconcentration maps.

## 3. Results

Situated at the transition from Pampean to Patagonian landscape, in geomorphological terms, the study area can be described predominantly as a plain characterized by a general level of planation [17], closely associated with the lithographic, structural, and sedimentary factors predominating in the region. A notable series of undulations and gently carved intermediate terraces descend stepwise from the plain to the narrow stretches of alluvial flats in the valleys that traverse the area from the northeast to the south and southwest in search of their base level, before eventually reaching the Atlantic coast at Bahía Blanca. A predominance of loess from the Pampean Formation intercalates with petrocalcic horizons, the relief varying from 210 to 0 m above sea level and the average general slope being 0.5%. 

**Figure 3:**
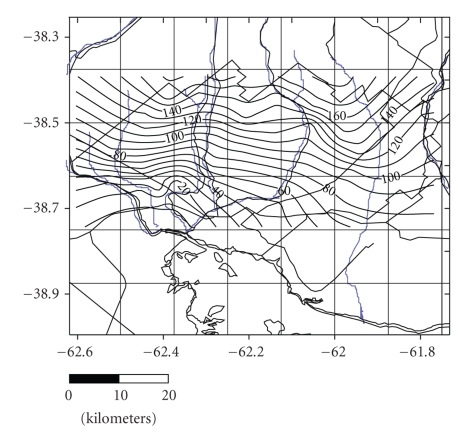
Isohypsic Chart.

The depth of the water in the wells and perforations varies from 1.2 m at the bottom of the valleys and close to the riverbeds to 55.8 m in the more elevated interfluve area. The morphology of the phreatic surface shows a notable symmetry, with a steep hydraulic gradient in the order of 4.5‰ in the western sector and 2.8‰ in the east, markedly parallel isohipses, and a clear orientation of the discharge towards the maritime coast (Figure 3). 

**Figure 4:**
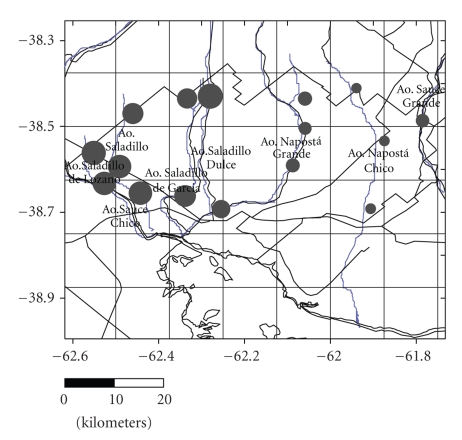
Magnitude of electrical conductivity (EC) in surface water

**Figure 5:**
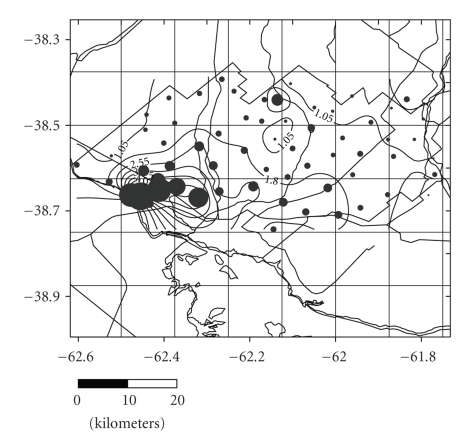
Isoconcentrations and magnitude of electrical conductivity (EC) in phreatic groundwater (wells).

In evaluating the results it was considered useful to have information on electrical conductivity since this parameter indicates increases in salinity as a function of increases in dissolved ion content, giving a clear diagnosis of the degree of salinity of the studied waters. The values observed for surface water were from 0.59 dS/m to 3.35 dS/m, the increase in salinity being particularly marked in water from the western sector of Bahía Blanca area (Figure 4). There was greater variability in the groundwater, showing a sequence of values from 0.30 dS/m to a maximum of 8.09 dS/m. The isoconductivity lines indicate the spatial behaviour of total soluble salts, showing a considerable increase in the concentrations in the extreme southwest of the area (Figure 5). 

**Figure 6:**
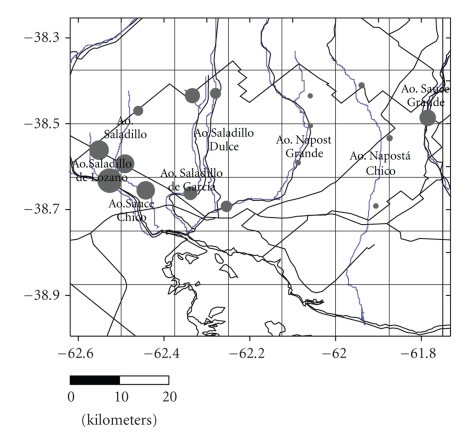
Magnitude of arsenic contamination in surface water.

The results from the chemical analysis of water (Tables 1(a), 1(b), and 1(c)) indicated that arsenic was detected in all the samples, the lowest values being found in 19 samples collected from surface water, with concentrations varying between 0.01 mg/L, the overall lowest value found in the study area, and a maximum of 0.13 mg/L (Figure 6). As in the case of electrical isoconductivity, the highest arsenic concentrations were found in the western sector of the study area. 

**Figure 7:**
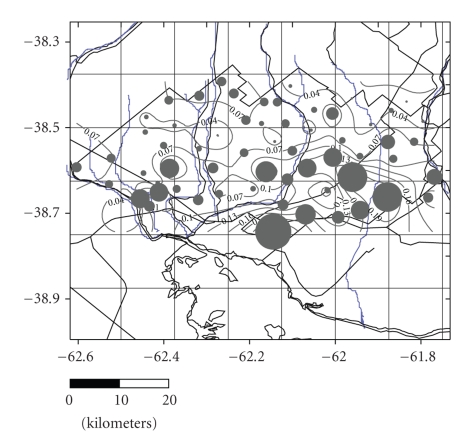
Isoconcentrations and magnitude of arsenic contamination in phreatic groundwater (wells).

**Figure 8:**
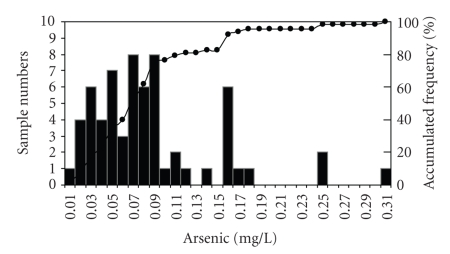
Frequency distribution of arsenic concentrations in groundwater. Data from samples collected June-July 2007.

Groundwater, the main source of supply for the rural population and for some suburban sectors, showed minimum concentrations of 0.01 mg/L and a maximum of 0.30 mg/L (Figure 7). The results indicate that concentrations increase towards the southwest, clearly following the direction of the flow towards the ocean. The results obtained for June–July 2007 (Table 2) show an irregular and asymmetric frequency distribution (Figure 8). The findings show the presence of varying concentrations of arsenic in a complex distribution pattern. 

The tolerance level recommended by the WHO [18] for this carcenogenic contaminant in drinking-water is 0.01 mg/L, this same value being cited in the Código Alimentario Argentino [19] and in force since 2007. On the basis of this criterion, only 3.2% of groundwater and 35% of surface water sources are within levels recommended for consumption. 

The presence of arsenic in the water samples collected from this region of Argentina is due to local geochemical conditions that facilitate the transfer of naturally occurring arsenic from soils and sediments to the water, favoured by zonal hydrodynamics [15]. No anthropic activities have been observed to contribute to the level of arsenic contamination in the water of the area.

## 4. Conclusions

The results of the current study indicate that approximately 97.0% of the water samples collected from groundwater wells and surface water sources used for consumption by humans and livestock exceed WHO guideline values for arsenic. These findings show that the population in the area may be exposed to the chronic toxicological effects of hydroarsenicism, placing them at greater risk of contracting a variety of illnesses. 

Surface water in the upper reaches of the riverbeds traversing the area is fit for human consumption. However, consumption of water from the riverbeds close to their outlet, where much higher concentrations of the contaminant and of total soluble salts were found, also poses a threat to public health. Since the groundwater studied here constitutes the principal source of drinking water in the zone, it is essential that the findings of this and similar research be made known and applied to practical solutions in preventive medicine, alerting the community to the potential danger associated with this contaminant. Although the government has installed a reverse osmosis plant for As-water treatment in the southwestern sectors of the studied area where in the highest population density is concentrated, the volume of water processed daily does not provide the demand for drinking water for the entire rural community. Nonetheless, this preventative measure sets up a very positive experience to reduce the risks to As-exposure through consumption of contaminated water. These treatment methods could be extended to other affected sectors of the southwestern pampa.

## Figures and Tables

**(a):** 

Latitude	Longitude	Depth (m)	Ce (dS/m)	As (mg/l)
S38 44 35.4	W62 08 40.9	18.3	1.54	0.30
S38 42 09.4	W62 04 09.8	15.0	1.91	0.17
S38 41 34.2	W61 56 31.0	13.4	1.46	0.15
**S38 41 29.9 **	** W61 54 22.4**	**—**	**0.61**	**0.01**
S38 39 43.7	W61 52 42.8	13.0	1.27	0.25
S38 39 48.1	W61 47 00.0	21.6	0.30	0.08
S38 36 54.3	W61 46 07.2	33.3	1.24	0.13
S38 31 58.8	W61 48 58.3	33.0	0.74	0.07
S38 34 22.1	W61 51 53.0	13.0	1.26	0.07
S38 36 56.3	W61 57 36.1	17.0	1.21	0.25
S38 38 46.9	W62 01 05.3	6.7	2.34	0.05
S38 32 00.7	W61 52 38.6	7.5	0.93	0.12
**S38 32 00.9 **	** W61 52 27.0**	**—**	**0.59**	**0.01**
S38 29 06.7	W61 47 43.7	4.7	0.66	0.02
S38 26 21.1	W61 50 01.7	12.6	1.53	0.02
S38 27 35.5	W61 52 10.7	6.7	0.55	0.04
**S38 24 37.8 **	** W61 56 21.6**	**—**	**0.53**	**0.01**
S38 25 54.0	W61 57 41.5	7.0	0.80	0.02
S38 29 34.3	W61 55 02.9	4.7	1.19	0.03
S38 31 46.8	W61 59 01.0	5.2	1.17	0.05
S38 34 10.2	W62 00 20.5	24.5	1.13	0.16
S38 40 48.1	W62 07 18.7	27.0	2.35	0.09
S38 39 17.0	W62 16 16.4	38.0	2.33	0.07
S38 28 01.6	W62 00 24.5	8.7	0.70	0.10
S38 24 09.9	W62 06 17.8	29.3	0.61	0.03
**S38 26 05.9**	** W62 03 33.0**	**—**	**1.27**	**0.01**
S38 27 30.2	W62 02 57.9	23.4	0.87	0.05
S38 30 20.6	W62 03 23.7	3.5	1.94	0.02

**(b):** 

Latitude	Longitude	Depth (m)	Ce (dS/m)	As (mg/l)
**S38 30 15.7 **	** W62 03 31.4**	**—**	**1.02**	**0.01**
S38 35 40.5	W62 03 56.4	24.3	1.60	0.16
**S38 35 26.0 **	** W62 05 15.5**	**—**	**1.22**	**0.01**
S38 37 15.3	W62 06 42.5	27.3	1.50	0.10
S38 38 33.7	W62 11 30.6	10.0	2.79	0.04
S38 33 14.8	W62 06 00.8	27.0	1.46	0.08
S38 31 56.8	W62 08 29.1	17.0	0.62	0.02
S38 29 25.6	W62 06 59.2	47.5	0.73	0.07
S38 29 25.2	W62 10 19.7	31.5	1.31	0.04
S38 26 26.0	W62 08 08.4	43.7	3.27	0.07
S38 25 13.1	W62 14 12.4	31.8	1.25	0.08
S38 28 57.5	W62 12 30.8	22.0	1.33	0.08
S38 26 24.1	W62 09 57.4	55.8	1.37	0.07
S38 33 31.0	W62 12 46.0	13.3	1.89	0.07
S38 36 11.4	W62 09 41.4	4.8	1.33	0.18
S38 35 39.2	W62 17 07.0	14.8	2.40	0.09
S38 31 10.0	W62 16 22.1	23.7	1.54	0.01
**S38 25 45.3 **	** W62 16 47.6**	**—**	**3.35**	**0.04**
S38 23 32.2	W62 15 54.7	23.0	1.41	0.08
S38 25 32.0	W62 19 02.5	6.7	1.32	0.08
**S38 26 04.5 **	** W62 20 01.8**	**—**	**2.42**	**0.07**
**S38 39 43.0 **	** W62 20 19.0**	**—**	**2.72**	**0.06**
S38 38 35.4	W62 22 12.5	10.0	4.96	0.06
S38 35 42.7	W62 23 12.3	1.8	2.77	0.16
S38 32 47.3	W62 23 19.5	20.0	1.35	0.06
S38 29 41.6	W62 22 30.3	27.0	1.24	0.03
S38 26 09.7	W62 23 19.0	26.4	1.22	0.07
S38 30 37.3	W62 26 36.3	16.4	1.08	0.04

**(c):** 

Latitude	Longitude	Depth (m)	Ce (dS/m)	As (mg/l)
S38 28 30.5	W62 26 30.4	11.3	1.06	0.04
**S38 28 11.1 **	** W62 27 36.6**	**—**	**2.59**	**0.04**
S38 39 57.7	W62 27 18.5	4.2	8.09	0.16
**S38 39 18.9 **	** W62 26 33.4**	**—**	**3.06**	**0.09**
S38 37 55.3	W62 31 40.6	2.0	1.81	0.07
**S38 37 57.4 **	** W62 31 36.2**	**—**	**3.03**	**0.13**
S38 35 33.1	W62 36 13.0	5.2	1.45	0.08
**S38 33 41.4 **	** W62 33 05.1**	**—**	**3.18**	**0.10**
S38 34 17.1	W62 31 22.7	21.7	0.69	0.07
**S38 35 35.7 **	** W62 29 28.0**	**—**	**3.03**	**0.09**
S38 36 22.4	W62 26 51.3	20.0	3.05	0.05
S38 37 44.7	W62 24 51.4	17.6	4.23	0.04
S38 40 08.3	W62 19 11.7	6.5	5.74	0.09
S38 32 57.1	W62 19 05.0	7.3	2.70	0.04
S38 34 00.1	W61 56 33.2	5.1	1.51	0.05
S38 42 33.8	W61 59 36.1	9.7	2.07	0.10
S38 39 04.0	W62 24 41.4	3.6	7.0	0.15
S38 39 47.0	W62 28 38.0	—	6.9	0.01
S38 39 56.6	W62 28 46.5	3.1	0.9	0.02
S38 40 50.4	W62 27 24.4	1.7	1.1	0.02
S38 41 00.7	W62 26 01.4	1.2	2.2	0.09
**S38 41 33.0 **	** W62 15 16.2**	**—**	**2.1**	**0.05**
**S38 29 10.7**	**W61 47 07.1**	**—**	**1.1**	**0.08**
**S38 42 06.8**	** W62 27 28.1**	**—**	**1.2**	**0.03**
**S38 33 29.9**	** W62 37 40.1**	**—**	**1.1**	**0.03**

**Table 2:** Summary results of arsenic content in phreatic groundwater-Data from samples collected June-July 2007

No. of samples	Minimum value	Maximum value	Mean	Standard deviation	Median	Range mode
	(mg/L)	(mg/L)	(mg/L)	(mg/L)	(mg/L)	(% samples)
63	0.007	0.302	0.081	0.060	0.070	0.07-0.09
						34.92%

## List of abbreviations and definition used

m: Metre
mg/L: Milligrams per litre
dS/m: Decisiemens per metre.
